# The neuronal Shc adaptor in Alzheimer’s Disease

**DOI:** 10.18632/aging.101368

**Published:** 2018-01-22

**Authors:** Viviana Triaca, Roberto Coccurello, Giacomo Giacovazzo

**Affiliations:** 1 Institute of Cell Biology and Neurobiology (IBCN), National Research Council (CNR), Rome, Italy; 2 European Center for Brain Research (CERC), Santa Lucia Foundation IRCCS, Rome, Italy

**Keywords:** neuronal Shc, neurotrophic signaling, brain cognition, neuroprotection, Alzheimer’s Disease

Preserving cognitive functions from age-related decline and Alzheimer’s Disease (AD) represents one of the major challenges of modern Neuroscience. However, we are currently unable to counteract the neurodegenerative process at an early stage, a prerequisite to minimize structural damage and recover learning and memory functions.

Reduced neurotrophic support by Nerve Growth Factor (NGF) and Brain Derived Neurotrophic Factor (BDNF) to the basal forebrain system has been extensively shown to cause cholinergic related cognitive dysfunction and neurogenesis alterations, thus contributing to AD aetiopathogenesis. Neurotrophin binding to the specific tyrosine kinase receptor (TrkA for NGF and TrkB for BDNF) allows phosphorylation and docking of early Trks adaptors shc (Src homology and collagen homology), thus promoting neuronal survival, phenotype maintenance and synaptic activity. Shc family is made of several isoforms sharing phosphotyrosine binding domains consisting of the N-terminal phosphotyrosine-binding (PTB) and the C-terminal Src-homology2(SH2) sequences, and is involved in brain development/functions, cancer and immunity.

**Figure 1:**
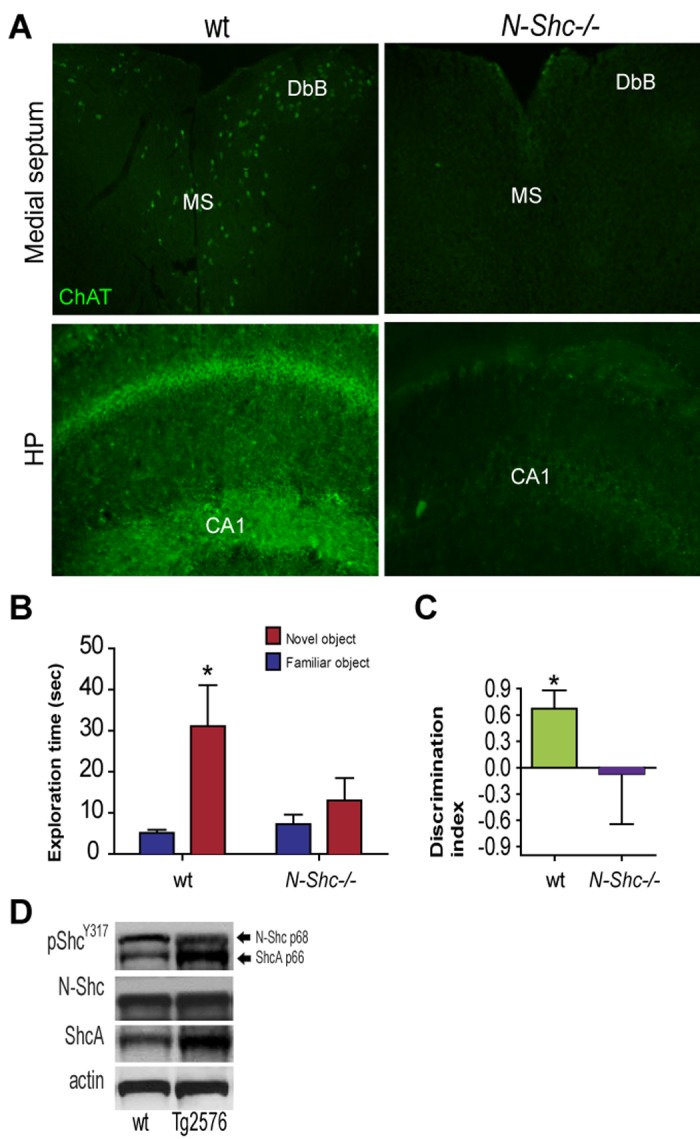
(**A**) ChAT fluorescent immunolabeling (Millipore, goat, 1:1000; anti-goat Alexa488 antibody) in the MS, DbB and CA1 of 10 months old male N-Shc ^-/-^ and age-matched male wt mice. (**B-C**) Adult (6 months old) male N-Shc ^-/-^ and age-matched male wt mice were subjected to vORT to test their cognition, by (**B**) the time exploring the two objects and (**C**) the Discrimination Index (DI; Normal cognition: DI>0.5). Data are expressed as means ±SEM. A *p < 0.05 difference was considered as statistical significant. (**D**) Western Blottings of septum extracts from 6 months old Tg2576 male mice were performed with specific antibodies against pShc^Y317^ (CST, 2431S, rabbit; 1:800), N-Shc (BD Transduction Lab, 610878, mouse; 1:1000), ShcA (mouse, 1:1000) followed by the respective secondary (anti-mouse or anti-rabbit) HRP-conjugated antibodies. Actin was used as loading control.

The neuronal Shc isoform C (N-Shc), also known as ShcC/Rai/Shc3/Shk, is envisaged as an elective molecular target to achieve brain neuroprotection in AD. The N-Shc protein evolutionary correlates with vertebrate brain development. In line with a major role in central nervous system functions, N-Shc is implicated in brain and retinal development, as well as in adult neurogenesis [1,2]. It is expressed as two 55 (p55) and 69 (p69) kDa isoforms specifically in post mitotic CNS neurons, and it is topographically enriched in the forebrain [3]. N-Shc initiates PI3 kinase-dependent sustained survival/differentiation, potentiates MAPK signaling and hampers JNK activation, being anti-apoptotic [4]. N-Shc activation exerts a crucial role in Long Term Potentiation (LTP) and is neuroprotective against aging-associated diseases, like ischemia [5] and oxidative stress [6]. In line with a major neuroprotective role of the signaling adaptor protein, a reduction of N-Shc protein and mRNA have been reported in brain aging, and AD [6]. Also, we observed a perturbation in the Shc pathway, with a specific down regulation of N-Shc phosphorylation in the forebrain of Tg2576 mice, a diffuse mouse model of AD (D). In line with this, N-Shc is necessary for the NGF control of Amyloid Precursor Protein (APP) metabolism in cholinergic neurons and, in turn, genetic reduction of N-Shc increases beta amyloid level [7]. A defective expression of ChAT, the specific cholinergic marker, occurs in the cell bodies of basal forebrain cholinergic neurons in the medial septum (MS) and Diagonal Band of Broca (DbB), and in their hippocampal target afferents in the Cornu Ammonis 1 (CA1) of N-Shc^-/-^ mice (Figure 1A). The cholinergic dysfunction may possibly result from NR2B persistent signaling induced by increasing amyloid level. Noteworthy, N-Shc null mice show recognition memory deficits, as seen by the visual Object Recognition Test (vORT). The time spent exploring the two objects (Figure 1B) and the Discrimination Index (DI; Figure 1C) have been assessed (Figure 1B-C). The wt mice spent a higher amount of time exploring the novel object (31.3±9.9 sec; *p<0.05), as compared to the familiar one (5.1± 0.7). N-Shc^-/-^ mice revealed significantly impaired novel object recognition, as shown by the comparable amount of time spent exploring the novel (13.0±5.5 sec) versus the familiar (7.3±2.3 sec) object. Accordingly, the DI of wt mice was in the normal range (DI: 0.67±0.08; *p<0.05), while N-Shc^-/-^ mice showed a total lack of discrimination between novel and familiar objects, (DI: -0.07±0.22).

N-Shc and its partners (e.g. GAB proteins) have been suggested to be key players in mammalian brain aging, cognitive longevity, and AD (Sagi et al., 2015). Of interest, the developmental isoform of Shc, namely ShcA, which is generated by alternative splicing of the *shc* gene, competes with the insulin/IGF metabolic pathway, potentially contributing to metabolic disturbances typical of the AD presymptomatic phase. Accordingly, our preliminary observations suggest that an N-Shc (p69) versus ShcA (p66) phosphorylation shift takes place, and total ShcA levels increase in the septum of AD mice (Figure 1D). Although caution should be taken for its potential cancerogenic effect, the selective expression/activation of N-Shc is a promising focus of future studies in the field and a novel target for septo-hippocampal neuroprotection in AD.

Collectively, current knowledge pinpoints N-Shc as a critical hub for neurotrophic pathways control of neuronal metabolism and phenotype/functions in the adulthood, of interest for AD and other metabolic and degenerative CNS diseases, like Parkinson’s disease and glaucoma.

Disturbances of neurotrophic signaling pathways have been extensively described as responsible for synaptic and metabolic derangements in neurodegenerative disease, like AD. Ineffective therapeutic interventions in AD have been attempted by targeting only one neurotrophic pathway at a time. The combined stimulation of NGF and BDNF pathways achieved by N-Shc targeting may hold the promise of a successful therapeutic approach. Here, we propose the design of molecules selectively targeting N-Shc to foster physiological and anti-amyloidogenic metabolism of basal forebrain cholinergic neurons, and to strengthen the septo-hippocampal system resilience in aging and AD neurodegeneration.
